# Replication of the natural selection of bad science

**DOI:** 10.1098/rsos.221306

**Published:** 2023-02-22

**Authors:** Florian Kohrt, Paul E. Smaldino, Richard McElreath, Felix Schönbrodt

**Affiliations:** ^1^ Department of Psychology, Ludwig-Maximilians-Universität München, Munich, Germany; ^2^ Department of Cognitive and Information Sciences, University of California, Merced, CA 95343, USA; ^3^ Santa Fe Institute, Santa Fe, NM 87501, USA; ^4^ Department of Human Behavior, Ecology, and Culture, Max Planck Institute for Evolutionary Anthropology, Leipzig, Germany

**Keywords:** agent-based model, replication, metascience, cultural evolution, incentives

## Abstract

This study reports an independent replication of the findings presented by Smaldino and McElreath (Smaldino, McElreath 2016 *R. Soc. Open Sci.* **3**, 160384 (doi:10.1098/rsos.160384)). The replication was successful with one exception. We find that selection acting on scientist’s propensity for replication frequency caused a brief period of exuberant replication not observed in the original paper due to a coding error. This difference does not, however, change the authors’ original conclusions. We call for more replication studies for simulations as unique contributions to scientific quality assurance.

## 1. Introduction

It has been argued that addressing the incentive structure of academia, which often rewards productivity and prestige over other more nuanced indicators of scientific quality, is key to supporting the credibility of published results [1]. One study underpinning this line of reasoning is a simulation by Smaldino and McElreath titled ‘The natural selection of bad science’ [2], in which the authors describe an evolutionary model of science. The aim of their study was to investigate the role of incentives for publication on the veracity, replication rate and methodological rigour of scientific publications, irrespective of individual researchers’ intentions.

The central finding of this study is that incentives to increase the number of one’s own publications can lead to an overall reduction in the quality of scientific research and an increase in the rate of false discoveries in the published literature. These findings also serve as argumentational base in numerous publications (e.g. [3–10]). The study reported here attempts to replicate the agent-based model presented by Smaldino & McElreath [2]. The replication was performed without knowledge or intervention from the original authors, which were involved in a later stage of the project to discuss the validity and the implications of the replication results.

The authors describe an evolutionary model of science, with individual laboratories as the unit of selection. In this model, laboratories repeatedly investigate hypotheses and attempt to publish their results. Laboratories can differ in three properties that characterize the full research process from hypothesis selection to making claims about its truth: the *replication rate* *r*_*i*_ describes the tendency of laboratory *i* to select an existing hypothesis for replication instead of coming up with a new one to test. Laboratories with a high *power*, *W*_*i*_, have a high probability of true and false positives, unless higher *effort*, *e*_*i*_, is exerted, which decreases the probability of false positives but also decreases a laboratory’s productivity (the rate at which it investigates new hypotheses). These three variables stochastically influence a laboratory’s publication quantity and subsequent pay-off, which ultimately determines whether their properties will be inherited by new laboratories. In evolutionary simulations, one or more of these properties were allowed to evolve through their associations with more successful laboratories, while other properties were held constant for the purpose of analytical clarity. Evolution worked through successful laboratories (those with more publications) being more likely to reproduce their methods in new laboratories, while the population size was held constant by the elimination of older laboratories at the same rate at which new laboratories were added.

The authors found that their simulated populations reliably evolved a decrease in effort and a rising proportion of false positives in the set of published results (false-discovery rate, FDR). They termed this central finding of their model *the natural selection of bad science*.

## 2. Method

In the context of simulation studies, a replication involves the creation of an independent implementation of the original conceptual model, based on the written algorithmic description. This can be done in the same programming language used to create the original implementation or in a different language. Results from the replication can be qualitatively and quantitatively compared with the originally reported results. This is different from a reproduction, which merely involves a re-run of the original source code. To protect against some biases, early exposure to the originally implemented model code was avoided by the programmer of the replication; only the fact that Java has been used as the programming language was known [11].

A first step of the replication was to write a concise specification of the original study’s *conceptual model*, which refers to the model formulation using text, equations and figures, but excluding the source code [11,12]. This specification was then used to implement an R package called labEvolution. The originally *implemented model*—i.e. the source code used in the original publication—was considered only after the first implemented replication.

Smaldino and McElreath illustrate their model’s results with five figures, which serve as reference for this replication attempt. A figure will be considered successfully replicated if *relational equivalence* is achieved. This replication standard means that both implemented models—both the original and the replicated versions—should show qualitatively similar relationships between input and output variables [13]. ‘For example, both models might show a particular variable as a quadratic function of time’ [13, p. 32]. Given the probabilistic nature of agent-based models, we would not expect strict numerical equivalence. This replication procedure draws from two widely used recommendations in the field [11,12].

The replication was implemented using the R programming language [14], and following best practices [15,16], the exact computational steps have been documented [17] along with the environment needed to reproduce them with one click [18,19]. The whole project has been archived in the Software Heritage source code archive [20] with additional metadata [21,22] concerning the citations [23] and licence terms [24].

## 3. Results

As in the original study, all results were averaged over 50 runs. Figures 3, 4, 6 and 7 of the original paper were successfully replicated based on the ‘relational equivalence’ criterion. Moreover, figs. 3 and 4 even showed numeric identity in their final values with a relative error of under 1%. Inconsistent results, however, were obtained for fig. 5 of the original paper that displays the coevolution of effort and replication rate. For the simulations reported in this figure, effort and replication rate were allowed to mutate, which meant their values could be inherited by child laboratories, while the laboratories’ power values were fixed to 0.8.

**Figure 1.**
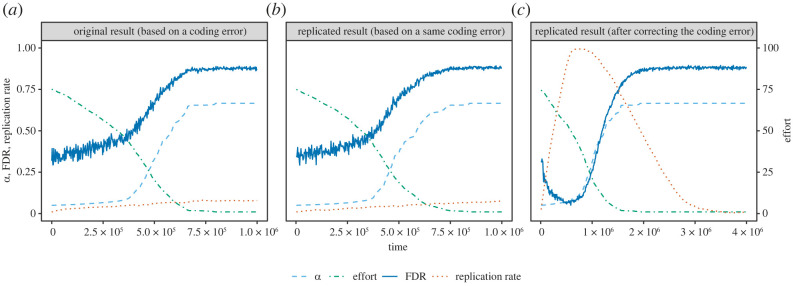
Comparison between the results of the original and the replicated model. Panel (*a*) is recreated from the data for fig. 5 of the original publication. This figure is available at https://doi.org/10.5281/zenodo.7547729 under a CC0 1.0 licence.

While effort, false positive rate (*α*), and FDR converge to the originally reported values when the simulated steps are extended beyond the original 1 × 10^6^ time steps, the replication rate’s progress and convergence are different from the original (see figure 1*a*,*c*).

The results of this replication show the following pattern: starting with a high-effort, low-effort replications are more attractive than conducting novel research (that is, employing this strategy received higher pay-offs), which results in the replication rate reaching nearly 100% after approximately 730 000 steps. At this point the decline of effort has made low-effort novel research more attractive than low-effort replications (because publishing a novel positive result is associated with a higher pay-off than publishing a replication) and consequently the replication rate decreases again. With the decline of effort, alpha rises up to 0.67, comparable to the value reported in the original study.

A comparison between the source code of the original published model and the replicated model revealed a coding error in the hypothesis selection mechanism that was able to fully explain the different results: according to the original model description, either a new hypothesis or an existing one is chosen, depending on the laboratory’s replication rate. However, the original software implementation mistakenly always uses the initial replication rate instead of the laboratory’s own replication rate *r*_*i*_.^1^ This has been corrected in the replication code.

Due to this bug in the original code, the replication rate could not influence the laboratories’ fitness, although it could mutate. The replication rate’s positive trend in the original study can be explained as an artefact of limiting the variable to the domain [0, 1] after mutation: random decreases greater than the initial value 0.01 will be bound to 0.01 in order to not become negative, but random increases will not be restricted until being very large (greater than 0.99, because the initial value 0.01 increased by more than 0.99 would exceed 1).

The R package that has been created as part of this replication allows recreating the original (erroneous) result as well, see figure 1*b*.

Another difference between the original model description and its source code, though we believe of minor importance, was the order in which laboratories are drawn from the population and chosen to reproduce during the stage *evolution*. According to the original model description, the dying laboratory is removed from the population before a laboratory is chosen to reproduce. The original code reverses this order, such that a laboratory may reproduce immediately before it is eliminated. This gives old, successful laboratories an additional chance to pass on their traits to new generations. However, a qualitatively different reproduction algorithm, in which laboratories were chosen for reproduction with a probability proportional to their pay-offs, has also been examined in the literature [9], and this did not qualitatively alter the results.

Finally, we note that section 5.3 of the original paper contains two typos concerning high and low efforts. The correct values are *e*_H_ = 75 and *e*_L_ = 15.

## 4. Discussion

The original study by Smaldino and McElreath described the conceptual model in sufficient detail to enable an independent implementation. Most of the study’s results could be replicated, regarding relational and close numerical equivalence. The exception was fig. 5, from which the authors concluded that the fact that the replication rate can evolve over time cannot ‘stave off the evolution of low effort’ [2, p. 12].

Although the specific evolutionary dynamics differ in the replication, the original conclusion, which was based on a coding error, remains unaffected by the replication’s corrected result. However, contrary to the description in the original study, effort initially decreases not despite the rise in replications, but specifically because low-effort replications are selected for. Our results imply that selection for productivity can temporarily incentivize replication if replication papers are easier to publish than novel results. Only when effort has sunk enough, low-effort novel research becomes more attractive and production of novel results will once again outcompete replication.

We would like to close with a call for reproduction and, in particular, for replication studies for agent-based simulations. We doubt that, in practice, simple code reviews would have caught coding errors like those detected in the present case. If full-blown reimplementations of simulation studies are necessary to evaluate their veracity and to substantiate their robustness, those efforts should be valued as original academic contributions. Through initiatives like RepliSims [25] and journals such as ReScience C [26] such endeavours can become more prevalent.

## Data Availability

Code and data of the replication have been archived in the Software Heritage archive [27].

## Footnotes

1 In the function definition of chooseHypothesis(AgentsSimulation) inside the file Boffin.java, line 86–102, the initial replication rate as.initReplication has been used, but instead the laboratory’s own property replicationProb should have been used.
